# Improved quantification of small hearts for gated myocardial perfusion imaging

**DOI:** 10.1007/s00259-013-2431-x

**Published:** 2013-04-30

**Authors:** Kenichi Nakajima, Koichi Okuda, Karin Nyström, Jens Richter, David Minarik, Hiroshi Wakabayashi, Shinro Matsuo, Seigo Kinuya, Lars Edenbrandt

**Affiliations:** 1 Kanazawa University Hospital, Kanazawa, Japan; 2 EXINI Diagnostics, Lund, Sweden; 3 Nuclear Medicine Unit, Skåne University Hospital, Lund University, Malmö, Sweden; 4 Clinical Physiology and Nuclear Medicine, University of Gothenburg, Gothenburg, Sweden; 5 Department of Nuclear Medicine, Kanazawa University Hospital, 13-1 Takara-machi, Kanazawa, 920-8641 Japan

**Keywords:** Myocardial perfusion imaging, Small heart, Left ventricular function, Software algorithm, Normal values

## Abstract

**Purpose:**

In patients with a small heart, defined as an end-systolic volume (ESV) of ≤20 mL calculated using the Quantitative Gated SPECT (QGS) program, underestimation of ESV and overestimation of ejection fraction (EF) using gated myocardial perfusion imaging are considered errors caused by inappropriate delineation of the left ventricle (LV). The aim of this study was to develop a new method for delineation of the LV and to evaluate it in studies using a digital phantom, normal subjects and patients.

**Methods:**

The active shape-based method for LV delineation, EXINI heart (ExH), was adjusted to more accurately process small hearts. In small hearts, due to the partial volume effect and the short distance to the opposite ventricular wall, the endocardial and the epicardial surfaces are shifted in the epicardial direction depending on the midventricular volume. The adjusted method was evaluated using digital XCAT phantoms with Monte Carlo simulation (8 virtual patients), a Japanese multicentre normal database (69 patients) and consecutive Japanese patients (116 patients). The LV volumes, EF and diastolic parameters derived from ExH and QGS were compared.

**Results:**

The digital phantom studies showed a mean ESV of 87 % ± 9 % of the true volume calculated using ExH and 22 % ± 18 % calculated using QGS. In the normal database, QGS gave higher EFs in women than in men (71.4 ± 6.0 % vs. 67.2 ± 6.0 %, *p* = 0.0058), but ExH gave comparable EFs (70.7 ± 4.9 % and 71.4 ± 5 % in men and women, respectively, *p* = ns). QGS gave higher EFs in subjects with a small heart than in those with a normal-sized heart (74.5 ± 5.1 % vs. 66.1 ± 4.9 %), but ExH gave comparable values (70.0 ± 5.9 % vs. 71.6 ± 4.2 %, respectively, *p* = ns). In consecutive patients, the average EFs with QGS in patients with ESV >20 mL, 11–20 mL and ≤10 mL were 57.9 %, 71.9 % and 83.2 %, but with ExH the differences among these groups were smaller (65.2 %, 67.8 % and 71.5 %, respectively).

**Conclusion:**

The volume-dependent edge correction algorithm was able to effectively reduce the effects on ESV and EF of a small heart. The uniform normal values might be applicable to both men and women and to both small and normal-sized hearts.

## Introduction

Electrocardiography (ECG)-gated myocardial perfusion single-photon emission computer tomography (SPECT) is a standard technology used today, and composite information on perfusion and function has been utilized for both diagnostic and prognostic purposes [1–3]. A number of studies since the 1990s have dealt with quantification of SPECT, and functional parameters such as ejection fraction (EF) and left ventricular (LV) volumes correlate well with those from left ventriculography, gated blood-pool studies and MRI. The interinstitutional reproducibility of these parameters is also excellent [4, 5]. However, it is well known that in subjects with a small LV volume LV end-systolic volume (ESV) is underestimated and EF is overestimated, and the errors are greater in women [6–12]. The so-called “small heart” effect has been found in various multicentre studies particularly in Japan. For example, in a multicentre investigation performed in Japan (J-ACCESS study), three-quarters of women with a low likelihood of coronary artery disease had a small heart [13]. This finding implies the necessity to use different thresholds between normal and abnormal subjects depending on the size of the heart.

The purpose of this study was first to develop a new method for delineation of the LV that more accurately quantifies small hearts. The new method was then evaluated using digital XCAT phantoms with Monte Carlo simulation, a normal database produced by the Japanese Society of Nuclear Medicine (JSNM) Working Group, and a clinical series of consecutive Japanese patients. EF, end-diastolic volume (EDV), ESV and volume curve differentiation (dV/dt) parameters calculated with the new method and with widely used cardiac quantification software were compared.

## Methods

### Definition of a small heart

In this study, a small heart was defined as a heart with an ESV of ≤20 mL as calculated using Quantitative Gated SPECT (QGS) software (Cedars Sinai Medical Center, Los Angeles, CA) [1, 14]. Patients with a small heart were further divided into two groups (SH20 group with ESV 11–20 mL and SH10 group with ESV ≤10 mL), and were compared with the group of patients with a normal-sized heart, i.e. LV with ESV >20 mL as calculated using QGS (NH group).

### Simulation study using digital phantoms

The SIMIND Monte Carlo program together with the XCAT phantom were used to generate projection data [15, 16]. A phantom representing a standard Japanese woman was assumed. Four different LV sizes with EDVs of 80, 60, 40 and 30 mL were used. For each LV size two different EFs of 65 % and 45 % were simulated, resulting in a total of eight different virtual patients. To simulate the beating heart and breathing motion, several static phantoms were created for each patient. A heart rate of 60 beats per minute and a 5-s breathing cycle were used. The entire breathing cycle was included, and the heart cycle was divided into eight frames, resulting in a total of 40 static phantoms, with the heart in five different positions for each heart frame. The five simulations for each heart frame were then merged. For each simulation, 32 projections in a 180° arc, starting at the 45° right anterior oblique position were created. A 128 × 128 matrix with a pixel size of 4.8 mm was used. In order to mimic real measurements, the simulations were performed with a sufficient number of photons to avoid Monte Carlo noise. Poisson noise was added after the simulations, corresponding to SPECT with an administered activity of 600 MBq, an uptake of 1 % in the heart and where every projection was measured for 20 s. Tomographic images were reconstructed using the ordered subsets expectation maximization algorithm with four iterations and eight subsets. A gaussian post-filter was applied with a full-width at half-maximum of 10 mm. Short-axis slices were created from the transaxial slices. Scatter and attenuation correction were not applied.

### JSNM database

A standard database created in 2007 by the JSNM Working Group for Standardization based on myocardial SPECT studies was used [17]. Patients with only 16 frames per beat were selected (69 patients; 33 women, 36 men). The database included only exercise stress–rest projection sets. The subjects had no ECG abnormalities indicative of ischaemia and no underlying cardiac disease. Subjects with hypertension and diabetes mellitus who required medication and with inappropriate arrhythmia for ECG gating were excluded. Both ^99m^Tc -labelled hexakis-2-methoxy-isobutylisonitrile (MIBI) and tetrofosmin were included. Wall motion determined by gated SPECT was considered to be normokinetic. Patients who had a normal coronary angiogram as well as those without an indication for coronary angiography because of a low likelihood of ischaemic heart disease were included. Low-energy collimators were used in all subjects. The acquisition angle for each projection was 4–6° per step with a rotation of either 180° or 360°. No attenuation correction was used in any of the hospitals. Patients with ESV <10 mL were excluded.

### Consecutive patients

Consecutive patients with suspicion of ischaemic heart disease and who were undergoing a stress–rest gated myocardial perfusion study were included. The studies were performed from October to December 2011. Of 116 patients, 79 men (mean age 59 ± 11 years) and 37 women (mean age 71 ± 11 years) were included. ^99m^Tc-MIBI and ^99m^Tc-tetrofosmin were used in 94 and 22 patients, respectively. The indications for stress myocardial imaging included evaluation of known coronary artery disease (45 %), screening for ischaemia due to multiple risk factors, preoperative evaluation of abdominal aortic aneurysm and peripheral vascular disease (38 %), chest symptoms and ECG abnormality (13 %) and other cardiac diseases including cardiomyopathies (4 %). The patients were classified into the three groups (NH, SH20 and SH10) based on the ESV determined by QGS software. The SPECT data were acquired with steps of 6° over 360°. ECG gating was performed with 16 frames per cardiac cycle. Since the purpose of the study was not to detect ischaemia, only resting gated SPECT data were used in the subsequent analysis.

### Data analysis

Gated SPECT data were processed using a standard software package to reconstruct short-axis images. Butterworth and ramp filters were used for filtered back projection reconstruction. To quantify LV function including EF, EDV and ESV, a standard method using QGS software was applied. QGS also calculates the dV/dt parameters as discussed below [14].

The new method for delineation of the LV, EXINI heart (ExH) software (Exini Diagnostics, Lund, Sweden), was based on a previously described method in which a heart-shaped LV model and an active shape algorithm are used instead of geometrical approximations such as ellipsoid or hybrid cylindrical/spherical models [18]. After automated location of the LV, the heart-shaped LV model is adjusted in an iterative process to optimize the fit of the mid-myocardial surface to the three-dimensional image data of the first frame. The endocardial and epicardial surfaces are defined symmetrically on each side of the surface defined by the maximal pixel count along each sampling profile perpendicular to the LV wall. A similar iterative procedure was applied to subsequent frames separately, i.e. no constraints regarding LV basal motion were included. Finally, the LV volume was calculated using the endocardial surface and the LV valve plane. Then EDV, ESV and EF were determined. After the volume curve was fitted by Fourier transformation, the dV/dt curve was created. The summation from the fundamental frequency to the third harmonic was used for the fitting of the original curve and calculated differentiation curve.

The method was adjusted to more accurately process small hearts. In small hearts, due to the partial volume effect and the short distance to the opposite ventricular wall, the ventricular volume appears to be smaller than the actual volume. Therefore, the endocardial and the epicardial surfaces are shifted in the epicardial direction before LV volumes are calculated. The size of the shift is calculated using the mid-ventricular volume of the LV using a univariate polynomial equation of the second degree. The equation was adjusted to give an increasing shift rate for decreasing volumes and a smooth transition to volumes above which no adjustment was performed. The parameters of the equations were determined empirically based on experience from a previous study [6] and analysis of myocardial perfusion studies not used in this study. The final equation was designed to produce a shift of 3.5 mm at a mid-ventricular volume of 0 mL and a decreasing shift up to a volume of 85 ml for which the shift was 0 mm. For mid-ventricular volumes greater than 85 mL, no adjustment was done.

Diastolic dV/dt parameters were calculated [14, 17, 19]. The peak filling rate (PFR) was defined as the maximum dV/dt value divided by EDV (per second). The one-third mean filling rate (1/3MFR) was calculated as the average of dV/dt values in the first third of the filling time (per second). The time to PFR (TPFR) was measured from end-systole to PFR (milliseconds). The ratio of TPFR (milliseconds) to the R-R interval (milliseconds) was also calculated.

### Statistical analysis

All data are expressed as means ± standard deviation (SD). Differences between groups were tested using the *t* test and analysis of variance. Differences in the data between the software algorithms were evaluated using a paired comparison test. Contingency analysis of categorical variables was also performed, and Fisher’s exact test and the Pearson test were used. Linear regression analysis of LV functional parameters was also performed. In the consecutive patients, comparison among multiple groups was performed for all pairs using the Tukey-Kramer method. A *p* value <0.05 was considered significant.

## Results

### Digital phantom

**Fig. 1:**
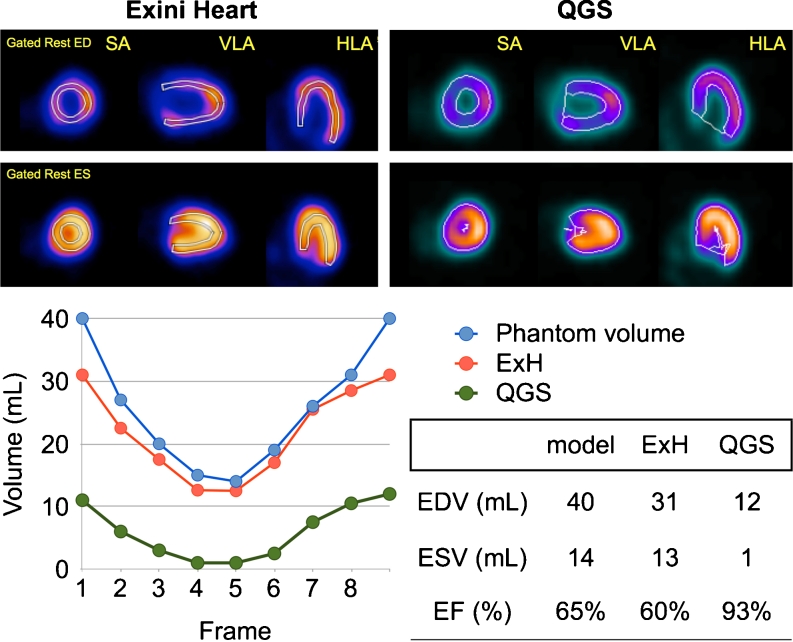
Digital phantom with EDV 40 mL and EF 65 %. Differences in edge tracing between ExH (*top left*) and QGS (*top right*) are apparent. Theoretical volumes of the phantom and those created by each software type are shown in the lower panels. *SA* short axis, *VLA*, vertical long axis, *HLA* horizontal long axis, *ExH* EXINI heart, *EDV* end-diastolic volume, *ESV* end-systolic volume, *EF* ejection fraction

The digital phantom images were processed using ExH and QGS. The LV delineations for the phantom with a true EDV of 40 mL and EF 65 % are shown in Fig. 1. The QGS software traced the endocardial borders into a small volume, but the border was traced near the mid-myocardial count by ExH. In relation to the true phantom volumes, ExH gave EDVs and ESVs of 74 % ± 5 % and 87 % ± 9 % and QGS gave values of 37 % ± 13 % and 22 % ± 18 % (Table 1). The EFs were always higher with QGS than with ExH.

**Table 1:** Digital phantoms and calculated EF and volumes

Model	Digital phantom			ExH					QGS				
	EDV	ESV	EF	EDV	Relative to true value (%)	ESV	Relative to true value (%)	EF	EDV	Relative to true value (%)	ESV	Relative to true value (%)	EF
1	30	10.5	0.65	24	80	7.4	70	0.69	6	20	0	0	0.93
2	30	16.5	0.45	24	80	16	97	0.33	6	20	1	6	0.80
3	40	14	0.65	31	78	13	93	0.60	12	30	1	7	0.93
4	40	22	0.45	31	78	19	86	0.39	13	33	4	18	0.67
5	60	21	0.65	43	72	20	95	0.54	26	43	4	19	0.83
6	60	33	0.45	43	72	29	88	0.32	27	45	12	36	0.56
7	80	28	0.65	53	66	25	89	0.53	41	51	11	39	0.73
8	80	44	0.45	54	68	34	77	0.37	42	53	21	48	0.50
Mean					74		87			37		22	
SD					5		9			13		18	

*EDV* end-diastolic volume, *ESV* end-systolic volume, *EF* ejection fraction, *ExH* EXINI heart, *QGS* quantitative gated SPECT software

### Normal database of the JSNM Working Group

In the normal database, a small heart with an ESV of ≤20 mL was observed in 17 of 33 women (52 %) and in 9 of 36 men (25 %; *p* = 0.027, Fisher’s exact test). None of the parameters (EF, ESV, TPFR and TPFR/RR) differed significantly between the two software programs (ExH and QGS, Table 2). ExH gave larger EDV, and smaller PFR and 1/3MFR than QGS. Normal values in men and women were compared (Table 3). EF determined using ExH did not differ between men and women, but using QGS was 4.2 % higher in women than in men (*p* = 0.0058). LV volumes determined using both methods were smaller in women than in men. The dV/dt parameters PFR and 1/3MFR determined using QGS were higher in women, but did not differ significantly with ExH.

**Table 2:** Paired comparisons between parameters determined using ExH and QGS in the JSNM normal database (values are means)

Parameter	ExH	QGS	Difference	*p* value
EF (%)	71.0	69.2	1.8	0.068
EDV (mL)	85.9	75.0	10.8	<0.0001
ESV (mL)	24.6	23.8	0.8	0.42
PFR (/s)	2.45	2.74	−0.29	<0.0001
1/3MFR (/s)	1.51	1.65	−0.14	<0.0001
TPFR (ms)	163.3	162.2	1.1	0.62
TPFR/RR	0.181	0.179	0.002	0.41

**Table 3:** Parameters determined using ExH and QGS in men and women in the JSNM normal database

Parameter	ExH			QGS			Paired *p* value (ExH vs. QGS)	
	Men (*n* = 36)	Women (*n* = 33)	*p* value	Men (*n* = 36)	Women (*n* = 33)	*p* value	Men	Women
EF (%)	70.7 ± 4.9	71.4 ± 5.0	0.54	67.2 ± 6.2	71.4 ± 6.0	0.0058	0.017	0.99
EDV (mL)	94.2 ± 15.7	76.7 ± 12.8	<0.0001	82.2 ± 17.5	67.2 ± 14.1	0.0002	<0.0001	0.0002
ESV (mL)	27.3 ± 4.7	21.7 ± 4.4	<0.0001	27.6 ± 10	19.7 ± 7.0	0.0003	0.84	0.11
PFR (/s)	2.45 ± 0.49	2.46 ± 0.64	0.96	2.56 ± 0.55	2.95 ± 0.74	0.015	0.056	<0.0001
1/3MFR (/s)	1.49 ± 0.38	1.55 ± 0.39	0.47	1.53 ± 0.37	1.77 ± 0.45	0.018	0.14	<0.0001
TPFR (ms)	175 ± 34	151 ± 27	0.0022	173 ± 34	151 ± 32	0.0080	0.45	0.96
TPFR/RR	0.186 ± 0.039	0.175 ± 0.020	0.12	0.183 ± 0.033	0.174 ± 0.022	0.19	0.32	0.91

The functional parameters in the NH and SH groups were compared (Table 4). EF determined using ExH did not differ significantly between the groups. EF determined using QGS, however, was 8.4 % higher in the SH groups than in the NH group. PFR determined using QGS was slightly higher in women than in men (*p* = 0.079), but it was similar with ExH. The dV/dt parameters 1/3MFR, TPFR and TPFR/RR did not differ significantly between the groups.

**Table 4:** Parameters determined using ExH and QGS in the NH and SH groups from the JSNM normal database

Parameter	ExH			QGS			Paired *p* value (ExH vs. QGS)	
	NH group (*n* = 43)	SH group (*n* = 26)	*p* value	NH group (*n* = 43)	SH group (*n* = 26)	*p* value	NH group (*n* = 43)	SH group (*n* = 26)
EF (%)	71.6 ± 4.2	70.0 ± 5.9	0.19	66.1 ± 4.9	74.5 ± 5.1	<0.0001	<0.0001	0.0034
EDV (mL)	91.2 ± 16.0	77.1 ± 14.4	0.0005	85.0 ± 13.6	58.5 ± 8.4	<0.0001	0.0050	<0.0001
ESV (mL)	25.8 ± 5.3	22.8 ± 4.9	0.024	29.2 ± 7.8	15.0 ± 3.5	<0.0001	0.0028	<0.0001
PFR (/s)	2.45 ± 0.50	2.46 ± 0.66	0.97	2.64 ± 0.60	2.93 ± 0.75	0.079	0.0035	<0.0001
1/3MFR (/s)	1.50 ± 0.35	1.53 ± 0.44	0.75	1.61 ± 0.41	1.71 ± 0.44	0.36	0.0016	0.0013
TPFR (ms)	166 ± 34	159 ± 32	0.37	165 ± 35	157 ± 32	0.33	0.75	0.71
TPFR/RR	0.180 ± 0.031	0.182 ± 0.034	0.75	0.178 ± 0.027	0.180 ± 0.032	0.77	0.53	0.60

**Fig. 2:**
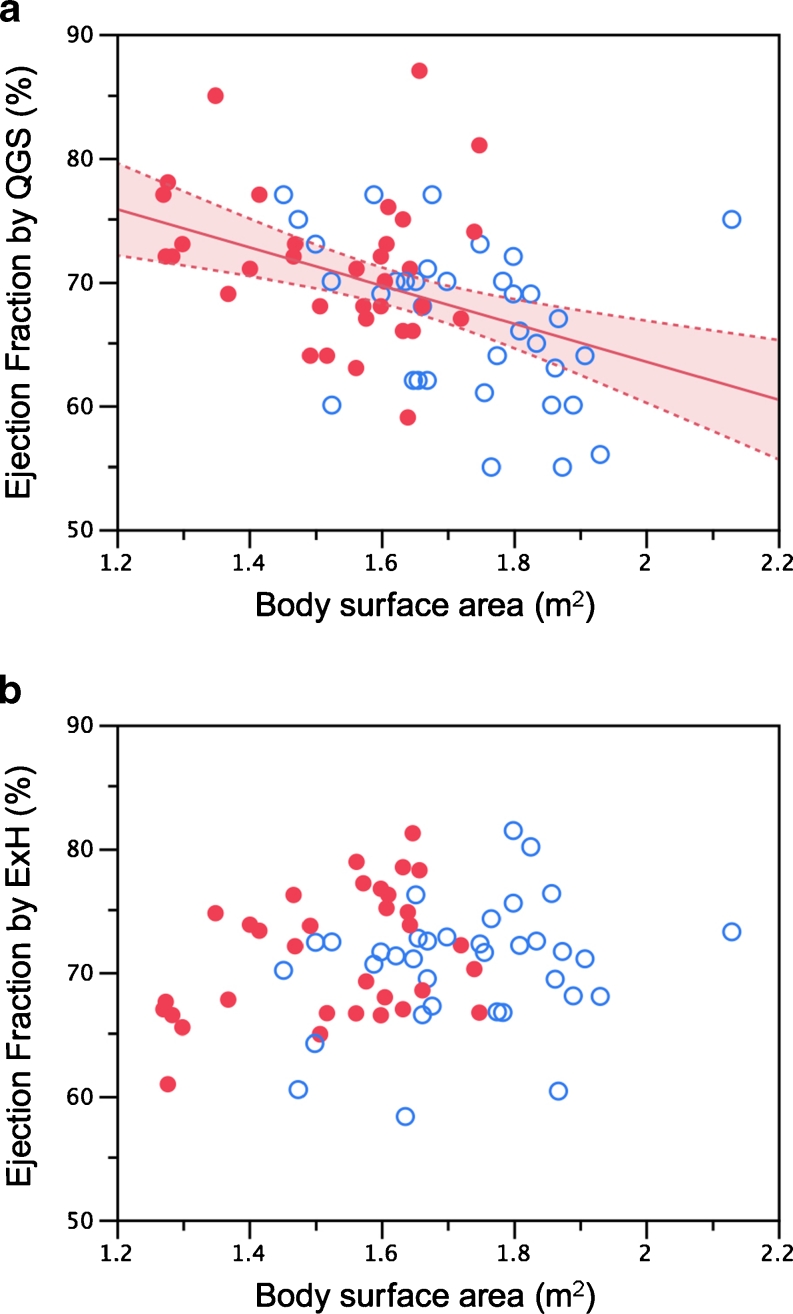
EF calculated using QGS (**a**) and ExH (**b**). The shaded area indicates confidence limits for the regression line (*red solid circles* women, *blue open circles* men)

EF determined using QGS was negatively correlated with body surface area (BSA, expressed in metres squared) (EF = 94.3 − 15.4 × BSA;* R* = −0.421, *p* = 0.0003), but no significant correlation was seen with ExH (*R* = 0.196, *p* = 0.11; Fig. 2).

### Consecutive patients

**Fig. 3:**
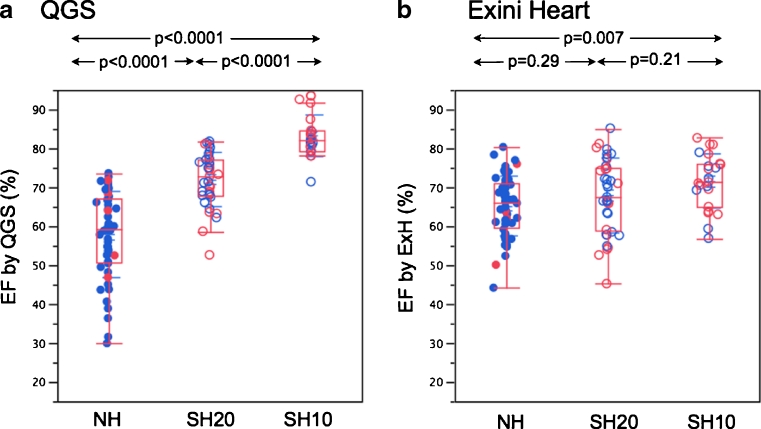
EFs of the NH, SH20 and SH10 groups of consecutive patients calculated using QGS (**a**) and ExH (**b**) (*solid circles* NH patients, *open circles* SH patients, *blue circles* men, *red circles* women)

**Fig. 4:**
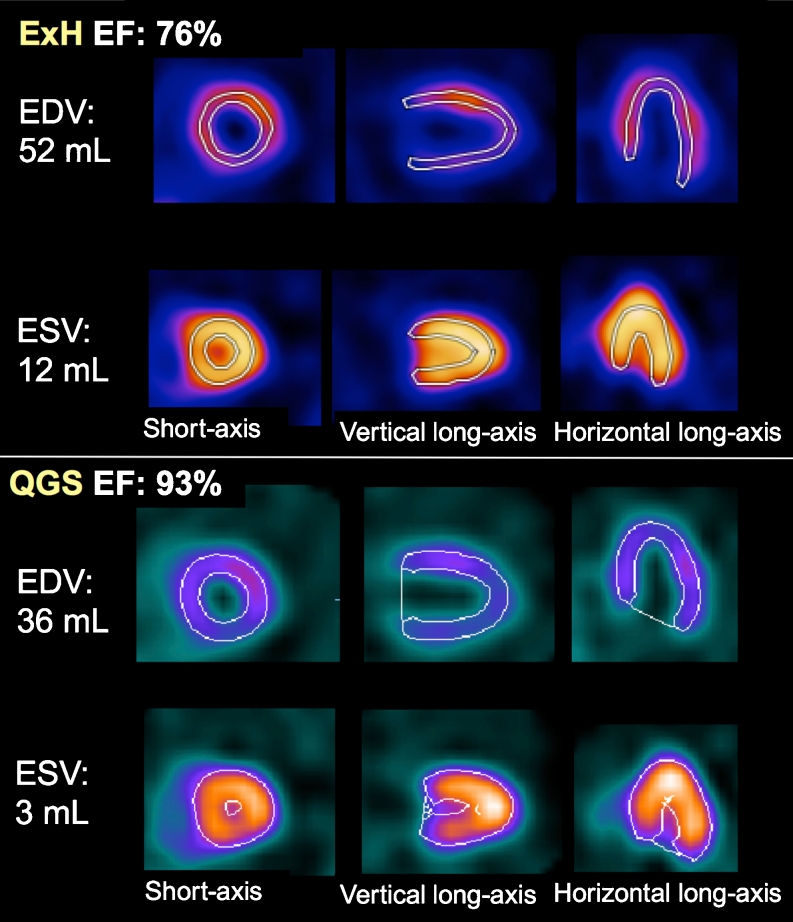
Preoperative gated SPECT imaging in a A 78-year-old woman with a small heart (height 142 cm, weight 43 kg, BSA 1.17 m^2^). The EFs calculated using ExH and QGS were 76 % and 93 %, respectively. Echocardiography showed EDV 64 mL, ESV 15 mL and EF 77 % by Teichholz’s formula

Among the 79 men, the NH, SH20 and SH10 groups comprised 50 (63 %), 21 (27 %) and 8 (10 %) patients, respectively, and among the 37 women the groups comprised 6 (16 %), 16 (43 %) and 15 (41 %) patients, respectively. Four patients who had an outlier EDV of ≥250 mL were excluded from the comparison of volumes and EF among the three groups (Fig. 3). Among the remaining 112 patients, 60 (54 %) had an ESV of ≤20 mL with QGS and 31 (28 %) with ExH (*p* = 0.0001, Fisher’s exact test). The average EFs in the NH, SH20 and SH10 groups were 57.9 %, 71.9 % and 83.2 % with QGS (*F* = 71.3, *p* < 0.0001), with a difference of 25.3 % between the NH and SH10 groups. However, using ExH, the average EFs were 65.2 %, 67.8 % and 71.5 % (*F* = 4.9, *p* = 0.01), with a difference of only 6.3 % between the NH and SH10 groups. An example of edge tracing in a patient with a small heart is shown in Fig. 4.

## Discussion

Inaccuracy of volume and EF calculations in patients with a small heart has been noted, but few solutions or practical correction methods have been proposed. We propose here a new method for LV delineation using a three-dimensional heart-shaped LV model, an active shape algorithm and a volume-dependent algorithm for delineation of the endocardial surface. The important advantage of this approach is that a uniform EF can be used in men and women as well as in small and normal-sized hearts.

The frequency of small hearts is relatively high in some populations. In the Japanese J-ACCESS population who showed a low likelihood of coronary artery disease and no cardiac events, a small heart was observed in 74 % of women and 13 % of men [13]. In an Iranian study, 85.4 % of subjects had ESV <25 mL, and most of them were women (112/123, 91 %) [20]. Although the frequency may differ among populations, the number of patients who may potentially require correction is not negligible. The finding that more than 80 % of patients have a suboptimal ESV indicates that the algorithm should be corrected rather than indicating a limitation of the software.

Digital phantoms were used to test the accuracy of volume determination. The digital phantoms were made so that clinical image characteristics were included. Although we created simple “cylindrical plus spherical” models in a previous study [6], the model used in this study was more realistic and was more reliable for validation. The ExH algorithm, however, still showed a slight underestimation of the volumes. The volume was strictly defined by the phantom, but due to some “blurring” effects caused by scatter, attenuation, depth-dependent resolution degradation, the beating of the heart and respiratory motion, to some extent underestimation of volumes could not be avoided. The volume of half a prolate spheroid with an equatorial radius of 25 mm and a centre to pole distance of 30 mm is 39 mL. If the radius is only 2 mm shorter, the volume is 31 mL, that is a 21 % underestimation. In this study, we could see significant improvement compared with the use of the QGS algorithm.

Normal values have been investigated in various populations including European, North American and Asian [11, 13, 14, 21–24]. In most of the studies, women showed EFs 5–11 % higher and smaller LV volumes than men. Differences between the EFs in men and women are relatively large in Japanese populations. The higher EFs in women are also associated with an EF limit that is as much as 10 % higher than in men. Although physiological differences between the genders might be one of the causes, the relatively higher frequency of small hearts might also be a factor. In this study after correction for the effects of small hearts, no significant differences, or at least only minor differences, were noted between the genders. In some multicentre studies, the threshold values for EF and ESV have been used as prognostic predictors. Therefore, a uniform EF that could be used in both genders and in small and normal-sized hearts would be useful for clinical practice and interinstitutional comparisons.

The dV/dt parameters are affected by the size of the heart. Diastolic parameters have been investigated in an American study, but subjects with a small heart (ESV<20 mL) were excluded [14]. However, filling rate parameters calculated using QGS showed higher values in women than in men. The PFR was also slightly higher in patients with a small heart. In contrast, the ExH algorithm gave comparable filling parameters between the genders and between normal-sized and small hearts. From a physiological point of view, higher rapid filling in small hearts and in women could not be explained, and was more likely due to a technical problem. Although the magnitude of dV/dt may have differed between genders, the timing parameters TPFR and TPFR/RR were identical between ExH and QGS software.

Some methods for overcoming the effect of heart size have been suggested. A high cut-off frequency of the Butterworth filter during processing could help improve visualization of the endocardial border [6, 8]. This approach has also been used in paediatric studies. However, changing the filter settings according to the chamber size is not practical for clinical studies, and the filter type used can affect the apparent myocardial tracer distribution in diseased hearts. Zoomed projection images during data acquisition has also been used. Zoom factors, such as 1.2 and 1.5 times, are possible. In clinical settings, however, changing the zoom factor before starting the study is not used in daily practice. Consequently, overestimation of EF and small ESVs are usually reported.

The effect of a small heart may differ depending on the algorithm used for ventricular edge determination [9, 10]. Common software types have individually developed algorithms to increase the correlation between EF and volumes determined by conventional methods and also to enhance the stability of edge tracing [25–27]. Although the precise steps in the algorithm have not been published, volume-dependent shifting of the edge detection algorithm has not been used. This study indicated the usefulness of volume-dependent correction since the effect of a small heart not only occurs below ESV <20 mL, but occurs gradually as the volume decreases [6].

The initial application of this algorithm to the clinical setting indicated that EF seemed to be more independent of ventricular volumes. In other words, when QGS software is used, whether or not the studied patients have a small heart should be considered to best judge the abnormality. Although additional studies are required, the results with the ExH algorithm indicate that the correction for a mean volume of <85 mL will work effectively in various populations.

There were some limitations to this study. The true volume was not determined from the patient data. Probably MRI might have been the first choice for this purpose, but it was not performed routinely in our hospital. Second, the algorithm for a small heart could have been improved by adjusting the range of volume for correction. Since the original images were blurred, various methods for improving the resolution might have been added, but this requires further investigation.

### Conclusion

The LV volume-dependent correction algorithm for subjects with a small heart provided a more uniform EF between genders as well as between small and normal-sized hearts. Normal values of EF and dV/dt variables were also stable. The application of this software to the clinical setting shows promise.
